# Ret is essential to mediate GDNF's neuroprotective and neuroregenerative effect in a Parkinson disease mouse model

**DOI:** 10.1038/cddis.2016.263

**Published:** 2016-09-08

**Authors:** Anja Drinkut, Karsten Tillack, Durga P Meka, Jorg B Schulz, Sebastian Kügler, Edgar R Kramer

**Affiliations:** 1 DFG Research Center Molecular Physiology of the Brain (CMPB), University Medical Center Göttingen, Göttingen, Germany; 2 Department of Neurodegeneration and Restorative Research, University Medical Center Göttingen, Göttingen, Germany; 3 Development and Maintenance of the Nervous System, Center for Molecular Neurobiology, University Medical Center Hamburg-Eppendorf, Hamburg, Germany; 4 Department of Neurology and JARA BRAIN Institute II, RWTH Aachen University and FZ Jülich, Aachen, Germany; 5 Department of Neurology, University Medical Center Göttingen, Göttingen, Germany; 6 Department of Applied Physiology, Ulm University, Ulm, Germany

## Abstract

Glial cell line-derived neurotrophic factor (GDNF) is a potent survival and regeneration-promoting factor for dopaminergic neurons in cell and animal models of Parkinson disease (PD). GDNF is currently tested in clinical trials on PD patients with so far inconclusive results. The receptor tyrosine kinase Ret is the canonical GDNF receptor, but several alternative GDNF receptors have been proposed, raising the question of which signaling receptor mediates here the beneficial GDNF effects. To address this question we overexpressed GDNF in the striatum of mice deficient for Ret in dopaminergic neurons and subsequently challenged these mice with 1-methyl-4-phenyl-1,2,3,6-tetrahydropyridine (MPTP). Strikingly, in this established PD mouse model, the absence of Ret completely abolished GDNF's neuroprotective and regenerative effect on the midbrain dopaminergic system. This establishes Ret signaling as absolutely required for GDNF's effects to prevent and compensate dopaminergic system degeneration and suggests Ret activation as the primary target of GDNF therapy in PD.

Glial cell line-derived neurotrophic factor (GDNF) is the founding member of the four ligands in the GDNF family, which belong to the transforming growth factor-*β* superfamily.^1^ GDNF was characterized as a potent survival factor for many neurons in culture such as dopaminergic, motor, sympathetic, parasympathetic, sensory and enteric neurons.^1, 2^ In addition, in dopaminergic neuron cultures GDNF stimulates neuronal differentiation, neurite outgrowth, synapse formation and dopamine release.^1, 2^

As degeneration of midbrain dopaminergic neurons in the substantia nigra pars compacta (SNpc) represents a major hallmark of Parkinson disease (PD), the most common neurodegenerative movement disorder, GDNF has raised considerable interest as a therapeutic molecule for the treatment of PD.^3, 4, 5^ PD affects >2% of individuals over the age of 60 years, but no curative treatment is available to date, mainly due to a lack of understanding disease etiology.^6, 7, 8^ Preclinical studies in the established 1-methyl-4-phenyl-1,2,3,6-tetrahydropyridine (MPTP) and 6-hydroxydopamine (6-OHDA) rodent and primate models of PD demonstrated a substantial neuroprotection and regeneration effect by striatal provided GDNF or its close relative neurturin.^3, 4, 9^ However, clinical phase II trials on PD patients using GDNF or neurturin did so far not convincingly recapitulate their beneficial effects on the dopaminergic system in humans most likely due to technical problems and the selection of advanced PD patients.^10, 11, 12, 13^

GDNF signaling is highly complex as this neurotrophic factor can bind to a variety of receptors, thus being able to induce pleiotropic effects. GDNF efficiently binds to the GPI-linked GDNF family receptor *α*1 (GFR*α*1).^1, 2^ It has been shown that the GDNF/GFR*α*1 complex can activate not only the canonical GDNF receptor Ret, a receptor tyrosine kinase which signals through the sarcoma protein (Src)/rat sarcoma (Ras)/mitogen-activated protein kinase (MAPK), phosphatidylinositol-4,5-bisphosphate 3-kinase (PI3K)/Akt, NF-*κ*B (nuclear factor 'kappa-light-chain-enhancer' of activated B cells), JNK (c-Jun N-terminal kinases) and PLC*γ* (phospholipase *γ*) pathway, but also with other signaling inducing receptors.^1, 2, 4, 5, 13^ So far, at least four alternative GDNF receptors have been described which are all expressed in midbrain dopaminergic neurons, NCAM,^14, 15^ the integrins *α*V and *β*I,^14, 16^ syndecan 3^17^ and N-cadherin.^18^ Interestingly, Ret is not essential during pre- and postnatal development of the mouse dopaminergic system,^19, 20, 21, 22, 23^ but specifically required for the maintenance of SNpc dopaminergic neurons and their striatal innervation in aged mice.^23, 24, 25^ In contrast, GDNF seems most likely under physiological conditions to be dispensable during development and maintenance of midbrain dopaminergic neurons in mice, although conflicting results exist.^26, 27, 28^ Thus, Ret might be activated by a GDNF-independent mechanism to stimulate SNpc dopaminergic neuron survival. In addition, the *in vivo* function of the alternative GDNF receptors in the dopaminergic system under physiological and pathophysiological conditions, like PD, and their dependence on GDNF has not yet been addressed in detail. This raised the important question which GDNF receptor might be required to mediate GDNF's reported neuroprotective and regenerative effect in the dopaminergic system in PD animal models and potentially in PD patients.^5, 29^

Previously, we showed in dopaminergic neuron-specific Ret knockout mice that Ret receptor loss does not result in a higher vulnerability of midbrain dopaminergic neurons against MPTP but to less resprouting of left over dopaminergic neuron axons in the striatum after MPTP intoxication.^30^ In adult mice endogenous GDNF levels are rather low.^26, 31^ Therefore, we could not rule out in that study the possibility, that higher levels of GDNF—as also used in the clinical GDNF trials in PD patients—might have neuroprotective and regenerating effects even in the absence of the Ret receptor. Here we addressed now this question by viral overexpression of GDNF in MPTP-treated mice lacking expression of Ret again specifically in dopaminergic neurons.^23, 30^ We found that in the absence of Ret in dopaminergic neurons even a substantial overexpression of GDNF in the striatum does not have a neuroprotective and regenerative effect. Thus, despite the expression of alternative GDNF receptors on midbrain dopaminergic neurons, the presence of the canonical GDNF receptor Ret seems to be mandatory for mediating GDNF's beneficial survival and axonal resprouting effect in these neurons.

## Results

**Figure 1:**
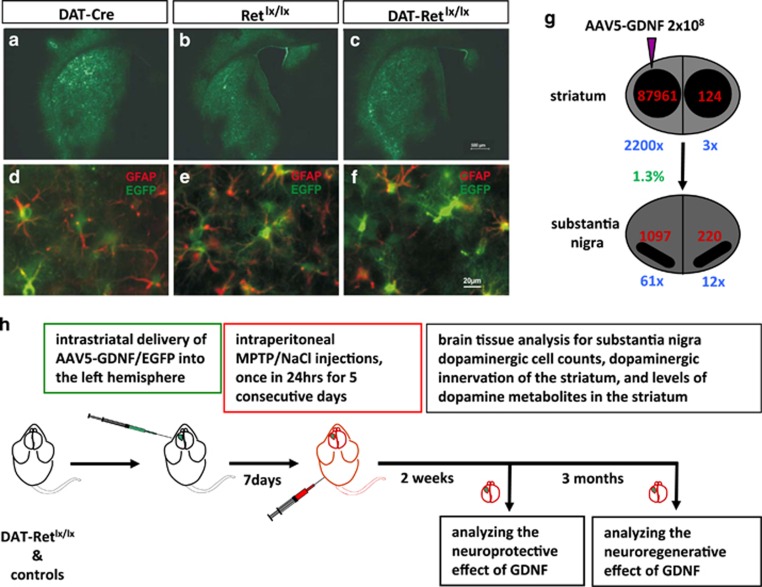
Tools and paradigm to address Ret function in mediating GDNF neuroprotective and regenerative effect in the MPTP mouse model of PD. (**a–f**) Coronal striatum sections are shown for the three mouse genotypes (DAT-Cre, Ret^lx/lx^ and DAT-Ret^lx/lx^) which were transduced with an adeno-associated virus of the serotype 5 encoding EGFP under the GFAP promoter to target astrocytes (AAV5-EGFP). (**a–c**) EGFP expression in the striatum of the left brain hemisphere at low resolution (scale bar, 500 *μ*m). (**d–f**) EGFP expression in green co-localizes in astrocytes immunohistochemically stained in red with antibodies against GFAP (scale bar, 20 *μ*m). (**g**) Schema shows absolute values of GDNF measured by ELISA in pg/mg tissue (red numbers) and fold of GDNF overexpression (blue numbers) of AAV5-GDNF injected mice in comparison to AAV5-EGFP injected wild-type animals with 40 pg GDNF/mg tissue in the striatum and 18 pg GDNF/mg tissue in the nigra. Only 1.3% of the GDNF expressed in the infected striatum is retrograde transported into the ipsilateral substantia nigra (green number). (**h**) Schema illustrating the experimental paradigm used

To investigate the Ret receptor function in mediating the neuroprotective and neuroregenerative effects of GDNF we generated mice with a disrupted Ret gene specifically in dopaminergic neurons (DAT-Ret^lx/lx^)^23^ by crossing mice carrying the floxed allele of Ret (Ret^lx/lx^)^32^ with dopamine transporter (DAT) promoter-driven Cre mice (DAT-Cre).^33^ We overexpressed GDNF in these mice by stereotactic injection of a recombinant adeno-associated virus serotype 5 (AAV5) encoding the mouse GDNF cDNA under the control of the glial fibrillary acidic protein (GFAP) promoter (AAV5-GDNF) unilaterally in the striatum of the left mouse brain hemisphere (Figure 1g and h).^34^ The contralateral hemisphere of the same animal served as an internal control as we found that this expression mode restricted the delivery of GDNF to only the vector-injected hemisphere (Figure 1g).^34^ Besides injecting a control virus encoding the enhanced green fluorescent protein under the GFAP promoter (AAV5-EGFP) in some mice confirming astrocyte-specific transgene expression, we also unilaterally sham-injected some mice (Figure 1a–f). Two weeks after injection of AAV5-GDNF we determined by immunohistochemical (IHC) and enzyme-linked immunoabsorbent assay (ELISA) the striatal GDNF levels to be 87 961 pg/mg tissue and the nigral GDNF levels of 1097 pg/mg tissue, whereas in AAV5-EGFP-injected animals striatal GDNF levels were 40 and 18 pg/mg tissues in the nigra. Thus, we achieved with the AAV5-GDNF vector an around 2200-fold and 61-fold overexpression of GDNF in the striatum and in the SNpc compared with AAV5-EGFP-injected mice, respectively (Figure 1g; Supplementary Figure 1). GDNF is spreading over the whole brain hemisphere sterotactically injected including the striatum and cortical regions (Supplementary Figure 1). The leakage of GDNF to the contralateral side is only threefold in the striatum and 12-fold in the substantia nigra (Figure 1g).

In our paradigm 1 week after virus injection the mice were challenged with a systemic subchronic MPTP treatment leading to 40–60% of dopaminergic system lesion or were treated with saline (NaCl) as a control (Figure 1h).^30^ The nigrostriatal dopaminergic system of these mice was analyzed 2 weeks later to monitor the neuroprotective effect of GDNF and three months after the MPTP treatment to investigate the neuroregenerative potential of GDNF (Figure 1h).^30^

**Figure 2:**
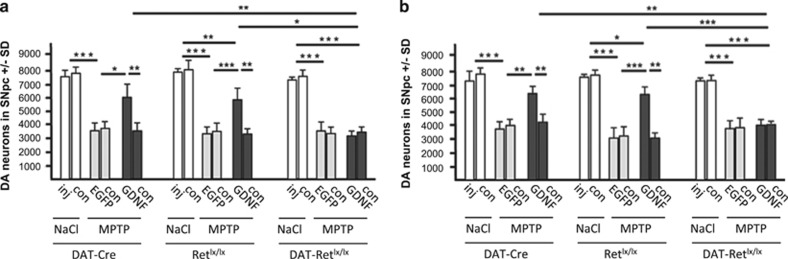
Stereological quantification of nigral dopaminergic neurons. Numbers of TH and Nissl-positive nigral neurons are shown for the three genotypes (Dat-Cre, Ret^lx/lx^ and DAT-Ret^lx/lx^) 2 weeks post MPTP treatment (**a**) and 3 months after MPTP treatment (**b**) and control mice which instead received saline (NaCl) (**a** and **b**). For each mouse the numbers on the side with mock injection (inj), AAV5-EGFP injection (EGFP) or AAV5-GDNF (GDNF) and on the non-injected contralateral side (con) are shown (*n*=6 animals per group)

First, we stereologically quantified the number of tyrosine hydroxylase (TH)-positive dopaminergic neurons of the SNpc in the mouse brains 2 weeks after MPTP treatment. In all three genotypes (DAT-Cre, Ret^lx/lx^ and DAT-Ret^lx/lx^) MPTP reduced the number of dopaminergic neurons by 46% (3000–4000 cells per brain half) compared with saline (NaCl)-treated mice (Figure 2a). Injection of AAV5-EGFP did not reduce MPTP-induced cell death in any genotype (Figure 2a). As expected, GDNF was neuroprotective in the AAV5-GDNF-treated brain hemisphere of control mice (DAT-Cre and Ret^lx/lx^) and MPTP induced <20% loss of dopaminergic neurons (Figure 2a). In contrast, GDNF expression conferred no neuroprotective effect against MPTP in Ret-deficient mice (DAT-Ret^lx/lx^). Thus, in the absence of Ret, even substantial amounts of GDNF did not protect dopaminergic neurons from MPTP toxicity. The same cell numbers were also observed in mice analyzed 3 months after MPTP treatment confirming the long-term neuroprotective effect of GDNF/Ret on dopaminergic neurons and also the general lack of dopaminergic cell regeneration after MPTP treatment independent of GDNF overexpression or not (Figure 2b). We did not observe a significant motor impairment in the MPTP-treated mice compared with the NaCl-treated mice using the open field, Rotarod and tight rope test, and could therefore not follow GDNF's beneficial effect on the behavior (data not shown). This is most likely due to <50% dopaminergic cell loss with the MPTP treatment (Figure 2).

**Figure 3:**
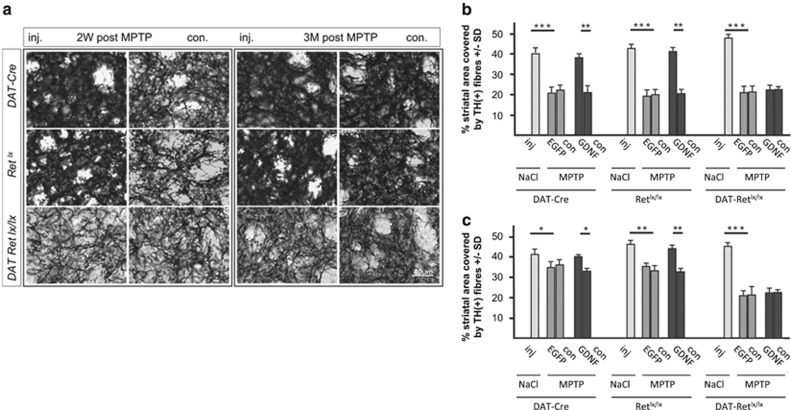
Quantification of striatal dopaminergic fiber density. TH immunoreactivity of striatal tissue sections is shown in **a** for DAT-Cre mice (upper panel), Ret^lx/lx^ mice (middle panel) and DAT-Ret^lx/lx^ mice (lower panel) at 2 weeks after MPTP treatment (first two columns) and 3 months after MPTP treatment (last two columns), for the injected hemispheres (inj., columns 1 and 3) and the contralateral hemispheres (con, columns 2 and 4). The quantification of striatal TH fiber density is presented in (**b**) for 2 weeks, and in **c** for 3 months after MPTP application. Scale bar, 40 *μ*m in (**a**) (*n*=6 animals per group)

Next, we investigated if GDNF's dopaminergic innervation protective function after MPTP treatment depends on the Ret receptor. Therefore, we assessed TH- and DAT- positive dopaminergic fibers in the striatum of the three genotypes 2 weeks and 3 months after MPTP intoxication (Figure 3; Supplementary Figures 2–5). Two weeks after MPTP treatment the striatal dopaminergic innervation was reduced by almost 50% in all three genotypes compared with NaCl-treated mice (Figure 3a). Injection of AAV5-EGFP had no influence on the MPTP-induced striatal dopaminergic axon loss (Figure 3a). Although the AAV5-GDNF-treated brain hemisphere of control mice treated with MPTP showed almost no degeneration of the striatal dopaminergic innervation, no protection was detectable in DAT-Ret^lx/lx^ mice (Figure 3b). Besides quantifying the 3,3-Diaminobenzidin (DAB) staining of the TH-positive fibers in the striatum (Figure 3), which does not allow to distinguish between alterations in TH protein and alterations in the amount of TH-positive fibers, we also performed a fluorescent TH staining and quantified precisely the relative density of dopaminergic fibers in the dorsal and ventral striatum using TH and DAT as markers (Supplementary Figures 2–5). These experiments support the DAB data.

**Figure 4:**
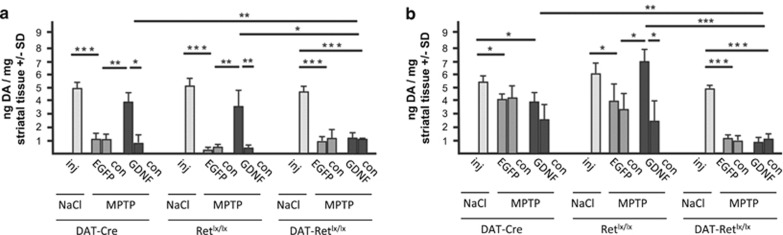
Quantification of striatal dopamine levels. Striatal dopamine levels assessed by HPLC are shown for control animals (NaCl), for animal receiving MPTP and AAV5-EGFP (MPTP+EGFP), and for animals receiving MPTP and AAV5-GDNF (MPTP+GDNF) for the vector injected (inj) and the contralateral hemisphere (con). Data are shown from the three genotypes (Dat-Cre, Ret^lx/lx^ mice and DAT-Ret^lx/lx^) at 2 weeks (**a**) and at 3 months post MPTP treatment (**b**) (*n*=6 animals per group)

To investigate the extent to which the residual dopaminergic fibers in the striatum remained functional, we quantified the total concentration of dopamine and its metabolites 3,4-dihydroxyphenylacetic acid (DOPAC) and homovanillic acid (HVA) in the striatum two weeks after MPTP treatment (Figure 4a; Supplementary Figure 4). Consistent with the dopaminergic fiber density also dopamine, DOPAC and HVA levels were only protected in the GDNF expressing hemisphere of DAT-Cre and Ret^lx/lx^ mice but not in DAT-Ret^lx/lx^ mice (Figure 4a). This shows that the protective function of exogenous GDNF on dopaminergic cell bodies and axons in the MPTP model depends on the presence of the Ret receptor in dopaminergic neurons.

Finally, we assessed GDNFs regeneration function at 3 months after MPTP in the three genotypes by quantifying TH- and DAT-positive fibers in the striatum (Figure 3c; Supplementary Figures 5 and 6). As observed previously,^30^ resprouting of the residual dopaminergic fibers after partial denervation by MPTP strictly depends on the presence of the Ret receptor and is already induced by endogenous levels of GDNF (about 0.04 ng/mg tissue; Figure 3c). Thus, substantial regeneration of dopaminergic fibers to 70–80% of saline injected control mice was detected in MPTP-treated DAT-Cre and Ret^lx/lx^ mice in the non-AAV-injected control brain site as well as in the AAV5-EGFP injected hemispheres, but not in Ret-deficient mice (Figure 3c). A significant additional regenerating effect of up to 90–100% of NaCl-treated control mice was detectable in the ipsilateral of AAV5-GDNF injected and MPTP-treated DAT-Cre and Ret^lx/lx^ mice (Figure 3c). But even after high- and long-term overexpression of GDNF no regeneration of dopaminergic fibers was detectable in the striatum of DAT-Ret^lx/lx^ mice (Figure 3c). The quantification of fluorescently labeled fibers reveals besides GDNF's neuroprotective effect also the significant resprouting of TH-positive fibers in the two control groups between 2 weeks and 3 months from 18 to 37% on the AAV5-GFP injected side of MPTP treated mice and 43 to 68% on the AAV5-GDNF injected side of MPTP treated mice compared with sham-injected mice (Supplementary Figures 2 and 4). We conclude that resprouting of dopaminergic axons after MPTP intoxication even in the presence of exogenous GDNF strictly depends on the presence of the Ret receptor. We also determined striatal levels of dopamine and its metabolites DOPAC and HVA by HPLC to rule out that GDNF might not influence the physiologically important neurotransmitter levels even in the absence of dopaminergic fiber regeneration in the Ret-deficient mice (Figure 4b; Supplementary Figure 4). However, in contrast to control mice where dopamine levels increased corresponding to the degree of regeneration no recovery of the dopamine levels took place in Ret-deficient mice demonstrating that also no functional recovery of the dopaminergic system is possible in the absence of Ret (Figure 4b).

To find out if our AAV5 vectors have a general effect on the brain physiology we also analyzed the glia response in our mice (Supplementary Figure 7). Although we did not find alterations in the number of microglia quantified with antibodies against ionized binding calcium adapter molecule (Iba)-1 in the SN and striatum, they led to a low, but persistent, increase in GFAP positive astrocyctes in the striatum 3 months after the MPTP treatment in addition to the previously described transient increase of astrocytes at two weeks after the MPTP treatment^30^ (Supplementary Figure 7). This mild gliosis seem not to be influenced by GDNF expression and seems also not to interfere with the observed beneficial effect of GDNF/Ret signaling on dopaminergic neuron regeneration.

## Discussion

We described in Kowsky *et al.*^30^ already that the absence of Ret does not decrease MPTP vulnerability leading to the same MPTP induced cell loss but prevents subsequent regeneration of dopaminergic fibers most likely mediated by endogenous GDNF. Here, in this manuscript, we overexpress now in addition GDNF before the MPTP treatment (Figure 1). This allows us to show in Ret expressing mice 2 weeks after MPTP treatment the neuroprotective effect of GDNF on the dopaminergic cell bodies and fibers in the striatum and additionally 3 months after MPTP treatment a more pronounced stimulatory effect of GDNF on regenerating dopaminergic fibers in the striatum (Figure 3; Supplementary Figures 2–5). Thus, indeed, we could show different effects of GDNF/Ret signaling on the midbrain dopaminergic cell body and the striatal dopaminergic innervation. The dopaminergic cell bodies require Ret for exogenous GDNF-induced protection from MPTP toxicity, although enhanced GDNF/Ret signaling cannot induce cell proliferation (Figure 2). In addition, Ret is required in the dopaminergic axons to mediate there exogenous GDNF's protective and regenerative effect (Figure 3; Supplementary Figures 2–5). This exogenous GDNF protects and also allows to partially recover the dopamine levels in the presence of Ret (Figure 4) suggesting for exogenous GDNF not only a beneficial effect on the fiber histology but also on the physiology. As the absence of Ret abolished already completely GDNF's neuroprotective function there was no need to apply GDNF after the MPTP induced degeneration to investigate only GDNF's regenerative function. Although the MPTP model of PD does not mimic all aspects of idiopathic PD,^35, 36^ it is a robust lesion model that allowed us to assess the requirement of Ret-mediated GDNF signaling. In our previous work we already established that Ret loss does not alter MPTP metabolism.^30^

Our result, that GDNF can provide its neuroprotective and neuroregenerative function on midbrain dopaminergic neurons only in the presence of the receptor tyrosine kinase Ret, has practical implications for therapeutic strategies to treat PD with GDNF and neurturin.^3, 7, 11, 12, 13^ It suggests that the main goal of clinical trials using GDNF and related substances should be to activate the Ret receptor. In our experiments even striatal GDNF levels 2000-fold above endogenous levels for >3 months were not sufficient to reveal any Ret-independent neuroprotective or regenerative effect of GDNF in the MPTP mouse model of PD. As the Ret receptor is only expressed in the dopaminergic system in dopaminergic neurons of the midbrain and GDNF expression was not able to suppress the mild but long-term gliosis in the SNpc, this supports the idea that GDNF stimulates directly intrinsic survival and resprouting pathways in dopaminergic neurons and additional effect on glia cells and other neurons seem less important. Thus, GDNF can only function efficiently when enough dopaminergic neurons are still around which express the Ret receptor.^5, 29^ Late stage PD patients with too few dopaminergic cells and strongly reduced striatal dopaminergic innervation will most likely not be able to respond efficiently to GDNF and should be excluded from the clinical GDNF trials. A PD therapy based on stimulating Ret signaling in dopaminergic neurons of the SN should be most effective in the early phase of the disease when there are still many dopaminergic neurons present on which Ret can mediate survival of dopaminergic neurons, protection from toxic insults, and resprouting of fibers in the striatum.^3, 7, 11, 12, 13^ Neurturin binds with high affinity on GFR*α*2,^1^ which is expressed in the ventral midbrain but not on midbrain dopaminergic neurons.^37, 38, 39^ This suggests that neurturin might stimulate Ret on dopaminergic neurons by low affinity binding to GFR*α*1 or supports dopaminergic neurons in a non-cell-autonomous manner.^39^

Interestingly, endogenous GDNF has been shown to be expressed in the adult striatum only in fast spiking, parvalbumin-positive GABAergic interneurons—representing 0.7% of all striatal neurons—and to a lower extent in some somatostatinergic and cholinergic interneurons, but not in medium spiny neurons.^13, 31, 40^ As GDNF cannot pass the blood–brain barrier (BBB), exogenous GDNF must be provided for the clinical trials by specific routes. GDNF protein can be intrastriatal or intranasal infused, modified or packaged GDNF that can pass the BBB might be supplied systemically, GDNF encoding DNA constructs might be provided by viral vectors, or GDNF expressing cells might be transplanted into the striatum.^3, 7, 11, 12, 13, 41^ Previously we compared astrocytic (human GFAP promoter) and neuronal (human synapsin 1 promoter) expression levels of GDNF with the AAV5 serotype vector and found similar GDNF expression levels in the injected side but less spreading to the contralateral side using the GFAP promoter.^34^ It has already been previously shown that AAV serotype 5 allows wide spreading of the virus in the striatum and efficient targeting of astrocytes.^34, 42^ The GFAP promoter led to high- and long-term GDNF expression in astrocytes without adverse effects as also shown previously in adeno- and lentivirus vectors.^34, 43, 44^ This is in contrast to high expression of GDNF in striatal and nigral neurons which can lead to negative side effects, such as downregulation of TH protein levels.^45, 46, 47, 48^ If in clinical trials the amount of bioactive GDNF in the striatum is limiting, the here used AAV5-GDNF serotype 5 vector with the GFAP promoter seems to be an efficient and safe alternative to provide GDNF.^34^ Further experiments are needed to clarify if the mild striatal gliosis found in our mice is also observed in humans and if this results in negative side effects.

Previous work has already shown that GDNF's neuroprotective function requires not only Ras-MAPK activation but also PI3K-Akt signaling.^5, 30, 49, 50, 51^ It is also well established that the Ret receptor it essential for maintaining aged SNpc dopaminergic neurons and seems to crosstalk for this maintenance function with proteins such as the chaperon DJ-1, PINK-1 (PTEN-induced putative kinase 1) and parkin, an E3 ubiquitin-protein ligase, for which mutated forms were found to lead to PD.^5, 24, 25, 29, 52, 53^ Ret and DJ-1 (deglycase) trigger dopaminergic cell survival in mice by activating together the Ras/MAPK pathway.^24^ Ret crosstalks with PINK-1 and parkin to ensure mitochondrial morphological and physiological integrity in dopaminergic neurons.^25, 52, 53^ Ret and parkin are not only important for survival of aged dopaminergic cell body in the SNpc but also for the maintenance of the striatal dopaminergic innervation.^5, 25, 29, 53^ Therefore, we suspect that these signaling events downstream of the Ret receptor play also an important role in mediating GDNF's neuroprotective and regenerative function in the dopaminergic system.

Although we have shown here the requirement for Ret for mediating GDNF's neuroprotective and regenerative effect in midbrain dopaminergic neurons, this does not rule out an additional function for alternative GDNF receptors in this context and also for mediating survival during development and maintenance of these neurons. The recent finding, that GDNF might be dispensable for the development and maintenance of the dopaminergic system in mice^26, 27, 28^ supports the idea, that alternative GDNF receptors might even function without the need to bind GDNF as important signaling recognition or cell adhesion molecules in dopaminergic neurons. Further studies are needed to clarify the physiological function of alternative GDNF receptors and GDNF itself in mice and man.

Small molecules that specifically activate Ret and can cross the BBB would be ideal and seem to be an achievable goal as small non-peptidyl quinol based GFR*α*1 agonists have already been developed.^54, 55^ Such substances would not only simplify the application procedure but would presumably also decrease the chance of unwanted side effects that might result from interaction with alternative GDNF receptors. If the Ret protein on dopaminergic neurons is limiting, Ret might also be provided by a viral vector approach targeting directly dopaminergic neurons.^10^ Such scenario has been created by high viral overexpression of *α*-synuclein in rats leading to downregulation of the orphan nuclear receptor Nurr-1 and its downstream target Ret.^56^ *α*-Synuclein is aggregating in Lewy bodies and neurites frequently observed in PD patients and gene amplifications and mutations of *α*-synuclein are found in some familiar forms of PD.^8, 57^ However, so far no^58^ or only mild^56^ reduction of Ret mRNA and protein levels have been found in PD patients, so that more investigations are needed to clarify this issue.

Taken together, our data support the ongoing clinical phase I trials on PD patients testing AAV-encoded GDNF and phase II trials using recombinant GDNF protein^12^ and emphasize the central role of Ret in mediating GDNF's survival and regeneration signal in SNpc dopaminergic neurons under neurodegenerative conditions that can cause PD.

It is likely that the beneficial effect of GDNF/Ret signaling might be further enhanced and more broadly applicable for the heterogeneity of idiopathic PD patients if combined with other neuroprotective and regenerative signals such as for example parkin overexpression.^29, 53^ Further experiments are needed to support this hypothesis.

## Materials and Methods

### Recombinant vector production

Recombinant AAV5 viruses expressing GDNF and EGFP were produced as described.^34^

### Animal procedures

Transgenic mice were kept on a C57Bl/6J background and were previously described.^23, 30^ All experimental animal procedures were conducted according to approved experimental animal licenses issued by the responsible animal welfare authority (Niedersächsisches Landesamt für Verbraucherschutz und Lebensmittelsicherheit) and controlled by the local animal welfare committee of the University Medical Center Göttingen.

Unilateral stereotactical injection of 3 × 10^9^ vector genomes (vg) of AAV5- GDNF or AAV5-EGFP in 2–3-month-old male mice and MPTP treatment was done as previously described.^34^ Animals were either sacrificed by cervical dislocation, the striata dissected, frozen and stored at −80 °C or animals were sacrificed using CO_2_ and after transcardial perfusion (10 min 0.1 M PBS, pH 7.4 and 10 min 4% PFA in PBS) the whole brain was fixed and cryoprotected (24 h in 4% PFA and 48 h in 30% sucrose at 4 °C).

### Detection and quantification of GDNF

GDNF was quantified by IHC staining and ELISA as described.^34^

### Histology

Stereological counting of dopaminergic neurons was performed by the optical fractionation method using StereoInvestigator as described.^30^ Glia cell and dopaminergic fiber density quantification in the striatum was performed as described.^23, 30^

Primary antibodies were used in the following concentrations: anti-TH (mouse, 1 : 2000, DiaSorin), anti-DAT (rat, 1 : 500, Millipore), anti-GFAP (rabbit, 1 : 300, DacoCytomation), and anti-Iba-1 (rabbit, 1 : 500, Wako).

### Quantification of dopamine, DOPAC and HVA

Catecholamine levels were determined by ion-pair HPLC with electrochemical detection essentially as described.^30^

### Statistical analysis

Data are expressed as means±S.D. To compare different group means for independent samples, the statistical analysis was performed by ANOVA, followed by Tukey's *post hoc* test (GraphPad Prism 4.0). Significance levels were set at *P<0.05, **P<0.01 and ***P<0.001.

## Abbreviations

DJ-1: deglycase, oxidative stress sensor and redox-sensitive chaperone and protease
GDNF: Glial cell line-derived neurotrophic factor
GFR*α*1: GPI-linked GDNF family receptor *α*1
MAPK: mitogen-activated protein kinase
NF-*κ*B: nuclear factor 'kappa-light-chain-enhancer' of activated B cells, transcription factor family with Rel homology domain
MPTP: 1-methyl-4-phenyl-1,2,3,6-tetrahydropyridine
PD: Parkinson disease
PI3K: phosphatidylinositol-4,5-bisphosphate 3-kinase
PLC*γ*: phospholipase *γ* cleaves the phospholipid phosphatidylinositol-4,5-bisphosphate (PIP2) into diacyl glycerol (DAG) and inositol 1,4,5-trisphosphate (IP3)
Ras: rat sarcoma; family of small membrane-associated GTPase
SNpc: substantia nigra pars compacta
Src: sarcoma protein; membrane-associated tyrosine kinase with different Src homology (SH) domains characteristic for all nine members of the Src family kinases
TH: tyrosine hydroxylase
