# Correction to: Ret is essential to mediate GDNF’s neuroprotective and neuroregenerative effect in a Parkinson disease mouse model

**DOI:** 10.1038/s41419-018-0636-4

**Published:** 2018-05-25

**Authors:** Anja Drinkut, Karsten Tillack, Durga P. Meka, Jorg B. Schulz, Sebastian Kügler, Edgar R. Kramer

**Affiliations:** 1 DFG Research Center Molecular Physiology of the Brain (CMPB), University Medical Center Göttingen, Göttingen, Germany; 2 Department of Neurodegeneration and Restorative Research, University Medical Center Göttingen, Göttingen, Germany; 3 Development and Maintenance of the Nervous System, Center for Molecular Neurobiology, University Medical Center Hamburg-Eppendorf, Hamburg, Germany; 4 Department of Neurology and JARA BRAIN Institute II, RWTH Aachen University and FZ Jülich, Aachen, Germany; 5 Department of Neurology, University Medical Center Göttingen, Göttingen, Germany; 6 Department of Applied Physiology, Ulm University, Ulm, Germany; 7 Present Address: Evotec AG, 22419 Hamburg, Germany

**Correction to**: *Cell Death Dis.* (2016) **7**, e2359; 10.1038/cddis.2016.263; published online 08 September 2016

Since the publication of this article, the authors reported that Edgar Kramer’s affiliation (labeled “6” in the affiliation notes) should read Plymouth University (rather than Ulm).

The authors apologize for any inconvenience caused.

