# Supplementary material for: Ret is essential to mediate GDNF's neuroprotective and neuroregenerative effect in a Parkinson disease mouse model
Source: Cell Death Dis. 2016 Sep 8;7(9):e2359–. doi: 10.1038/cddis.2016.263 (PMC5059866; doi:10.1038/cddis.2016.263)

Supplementary Figure-1 Kramer

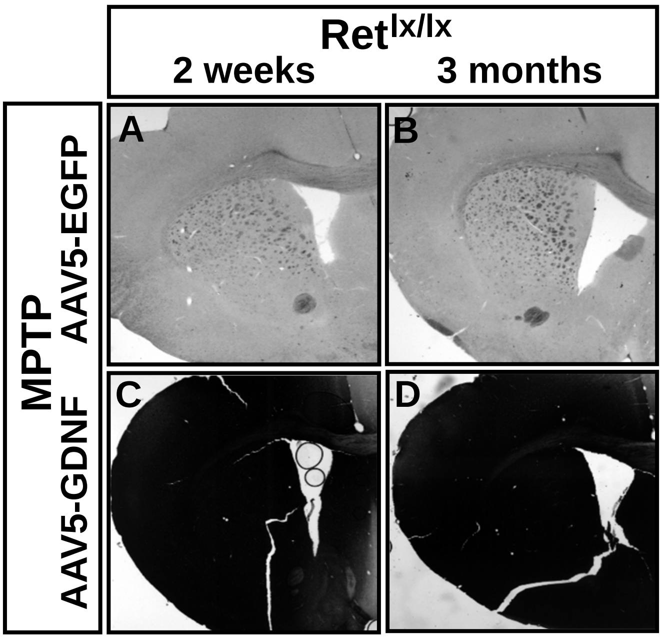

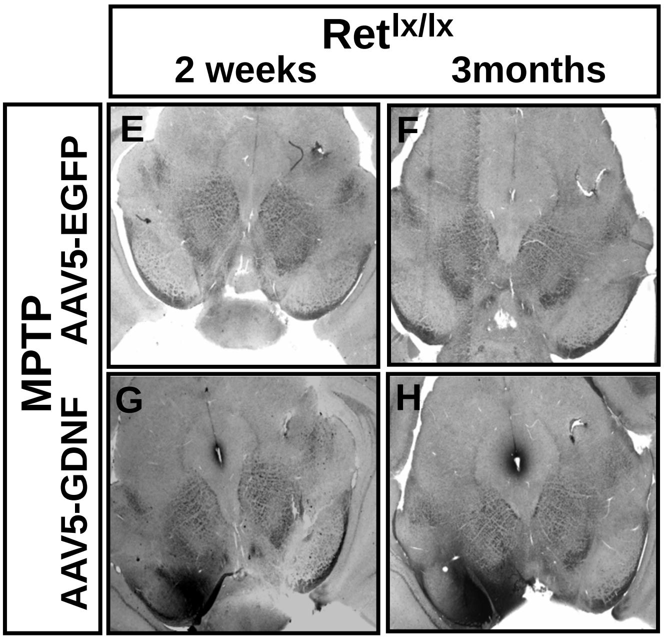

Supplementary Figure-2 Kramer

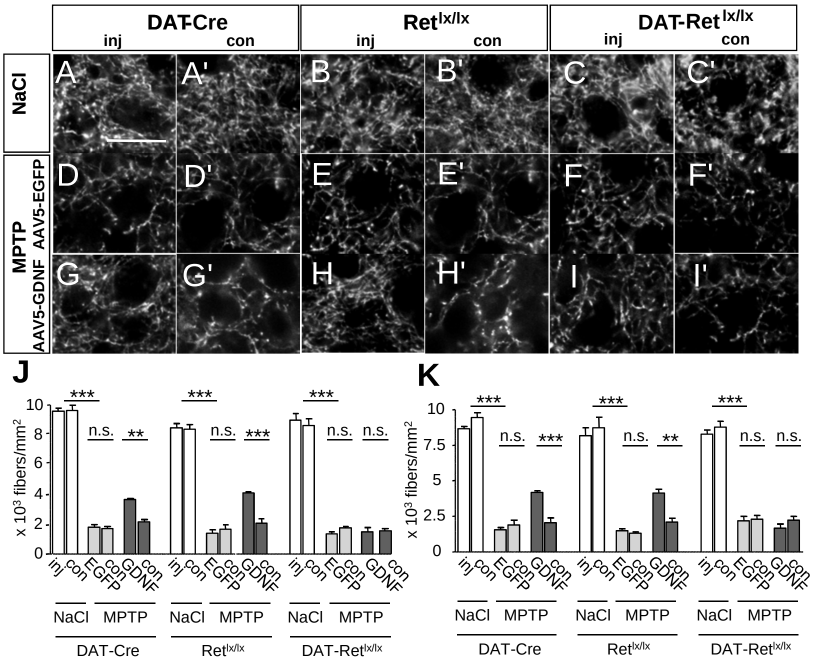

Supplementary Figure-3 Kramer

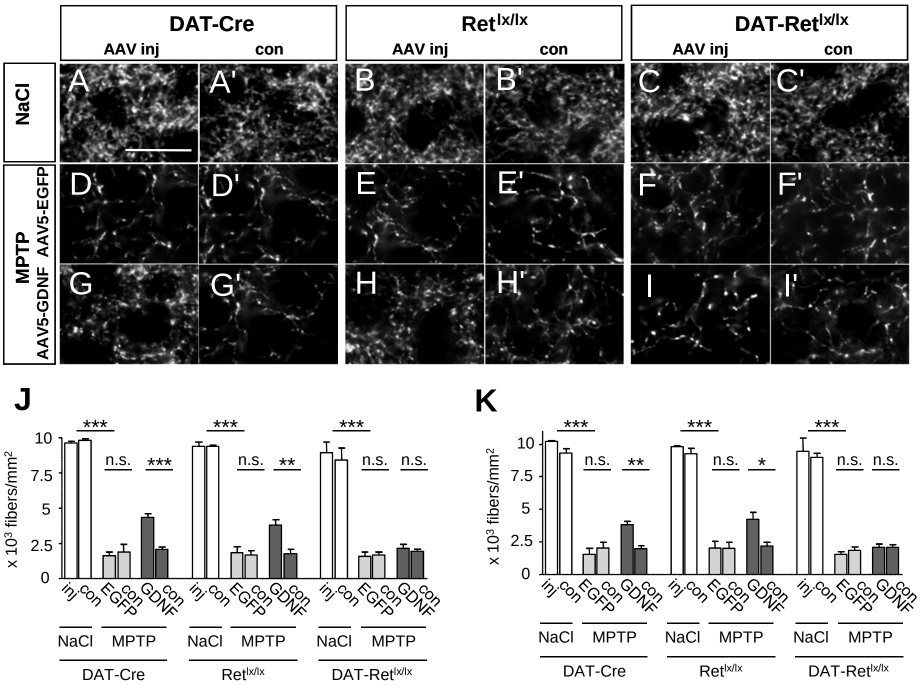

Supplementary Figure-4 Kramer

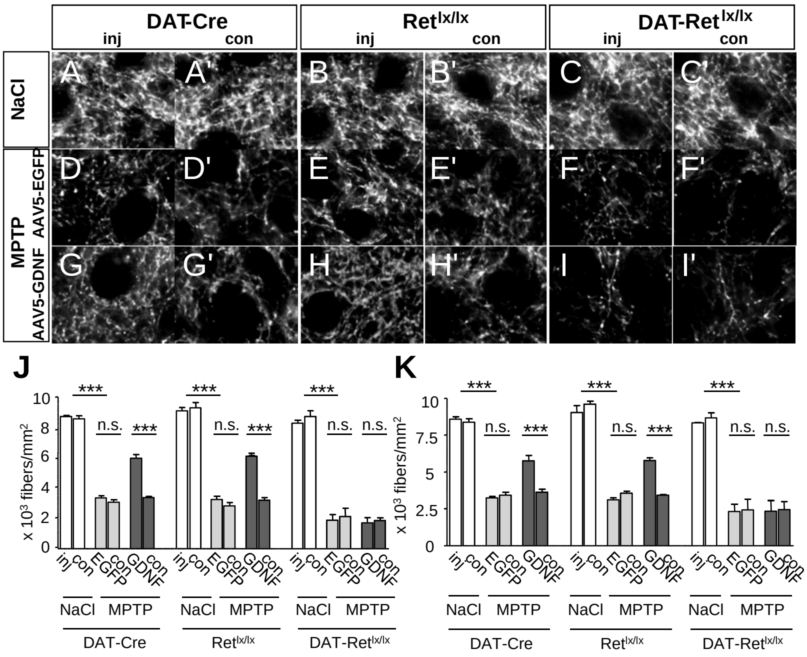

Supplementary Figure-5 Kramer

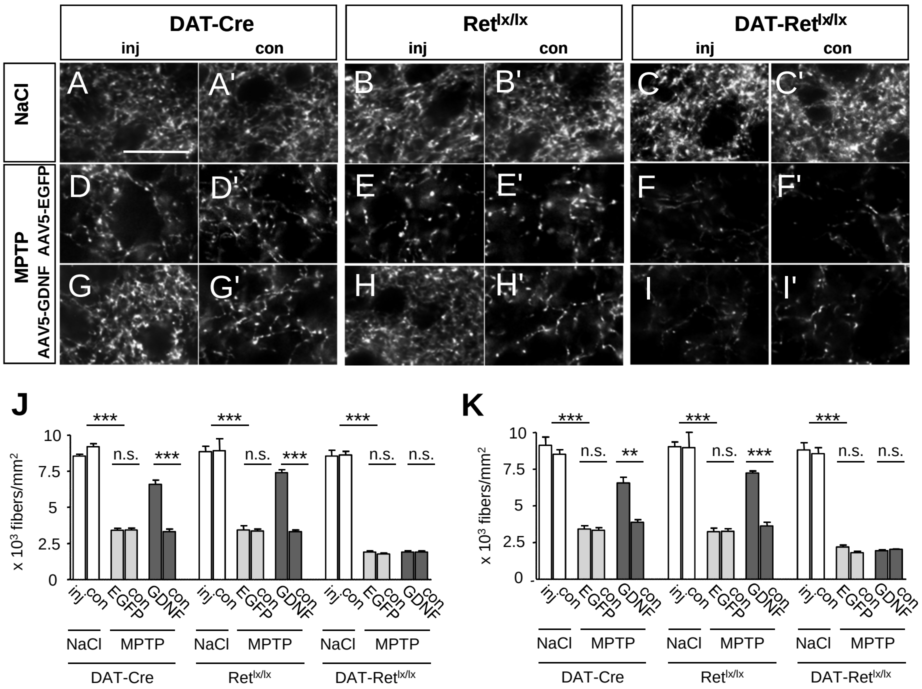

Supplementary Figure-6 Kramer

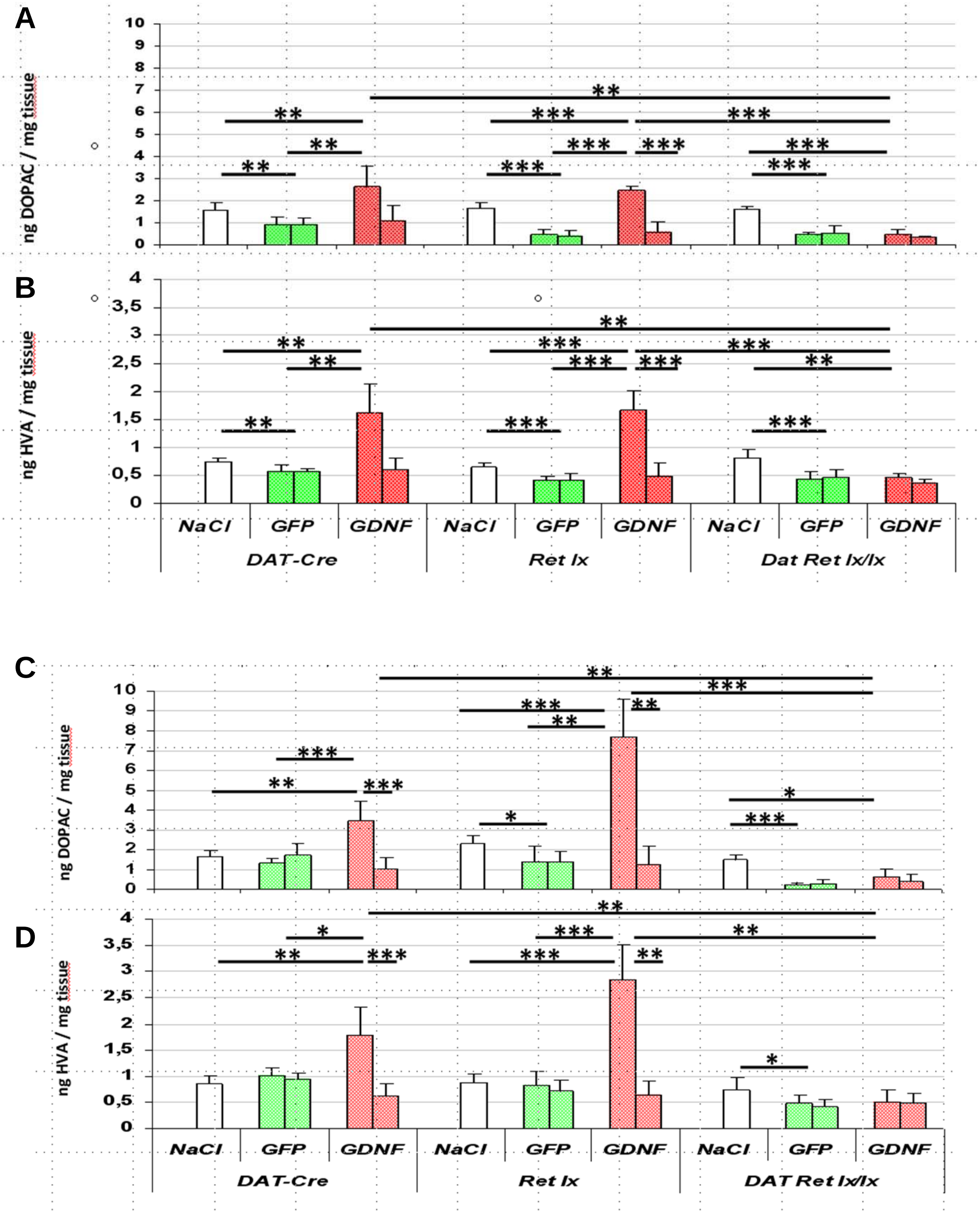

Supplementary Figure-7 Kramer

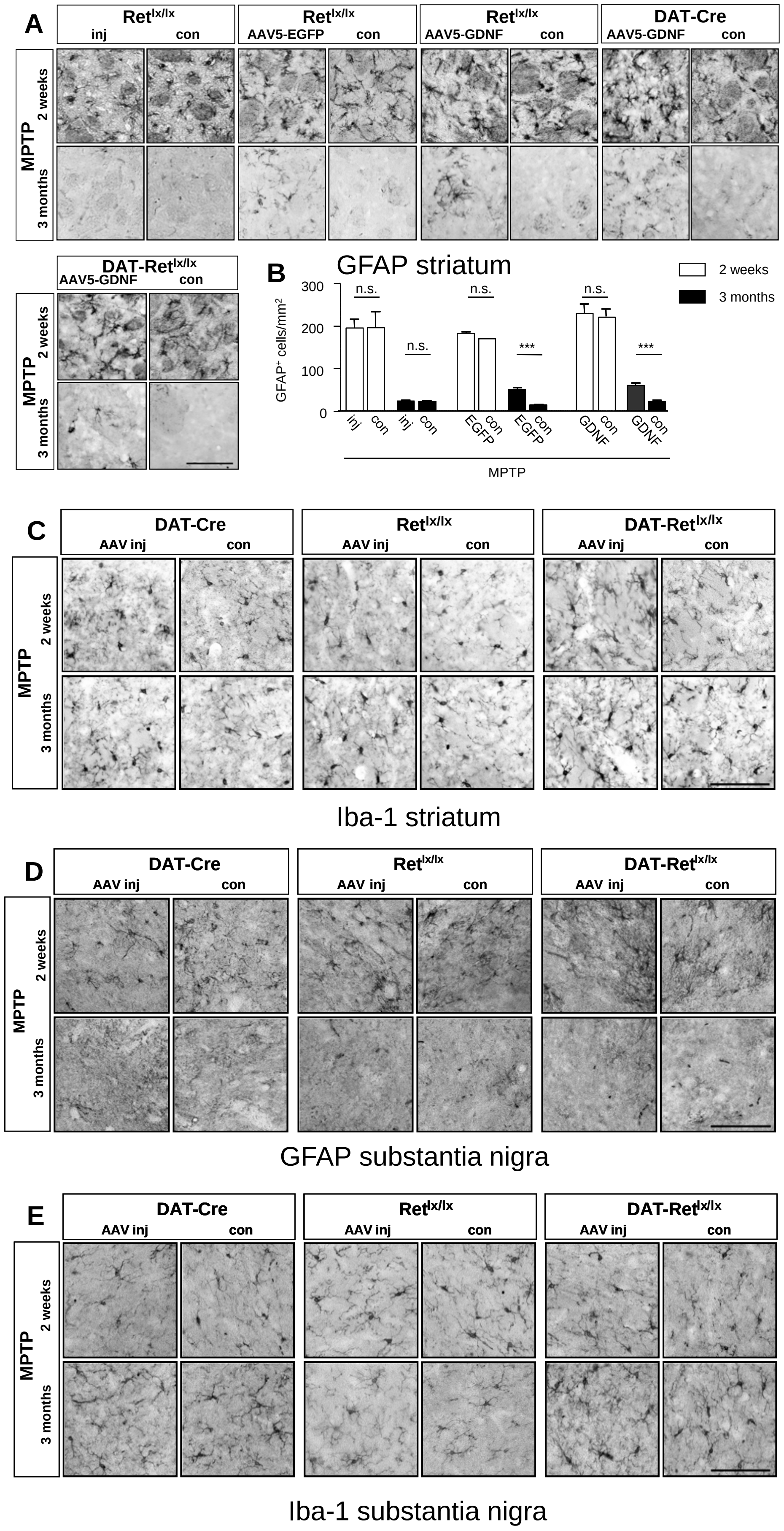

Supplement: Supplementary Figures [file cddis2016263x1.pdf]
