# Supplementary material for: Ret is essential to mediate GDNF's neuroprotective and neuroregenerative effect in a Parkinson disease mouse model
Source: Cell Death Dis. 2016 Sep 8;7(9):e2359–. doi: 10.1038/cddis.2016.263 (PMC5059866; doi:10.1038/cddis.2016.263)
Supplement: Supplementary Figure Legends [file cddis2016263x2.doc]

**Supplementary Figure legends:**

**Supplementary Figure 1: Efficient GDNF expression in the striatum of AAV5-GDNF treated mice**

Immunohistochemical staining for GDNF in coronal striatal (A-D) and substantia nigra (E-H) sections from control mice (Ret^lx/lx^) two weeks and three months after striatal injection of AAV5-EGFP (A,B,E,F) or AAV5-GDNF (C,D,G,H). There is only a strong GDNF signal in the AAV5-GDNF (C,D), but not in the AAV5-EGFP injected striatum (A,B). GDNF is also efficiently detectable in the ipsilateral but not contralateral substantia nigra of AAV5-GDNF injected mice (G, H).

**Supplementary Figure 2: Neuroprotective effect of exogenous GDNF on the striatal dopaminergic innervation detected by antibodies against tyrosine hydroxylase (TH)**

Protection of the dopaminergic system in the striatum was assessed two weeks after MPTP or saline treatment by quantifying the TH-positive fibers in the dorsal (A-J) and ventral striatum (K). The data confirm the results of the TH staining intensity in the striatum presented in Figure 3. Scale bar = 20 µm; n ≥ 3.

**Supplementary Figure 3: Neuroprotective effect of exogenous GDNF on the striatal dopaminergic innervation detected by antibodies against dopamine transporter (DAT)**

Protection of the dopaminergic system in the striatum was assessed two weeks after MPTP or saline treatment by quantifying the DAT-positive fibers in the dorsal (A-J) and ventral striatum (K). The data confirm the results of the TH staining intensity in the striatum presented in Figure 3. Scale bar = 20 µm; n ≥ 3.

**Supplementary Figure 4: Neuroregenerative effect of exogenous GDNF on the striatal innervation detected by antibodies against tyrosine hydroxylase (TH)**

We assessed regeneration of the dopaminergic system in the striatum three months after the MPTP or saline treatment by quantifying the TH-positive fibers in the dorsal (A-J) and ventral striatum (K). The data confirm the results of the TH staining intensity in the striatum presented in Figure 3. Scale bar = 20 µm; n ≥ 3.

**Supplementary Figure 5: Neuroregenerative effect of exogenous GDNF on the striatal innervation detected by antibodies against dopamine transporter (DAT)**

We assessed regeneration of the dopaminergic system in the striatum three months after the MPTP or saline treatment by quantifying the DAT-positive fibers in the dorsal (A-J) and ventral striatum (K). The data confirm the results of the TH staining intensity in the striatum presented in Figure 3. Scale bar = 20 µm; n ≥ 3.

**Supplementary Figure 6: Measurement of dopamine metabolites in the striatum**

Levels of the dopamine metabolites DOPAC (A,C) and HVA (B,D) were determined in the striatum of the mice two weeks (A,B) and three months (B,D) after MPTP or saline treatment. GDNF expression significantly increased DOPAC and HVA levels above normal levels in saline-treated control mice (DAT-Cre, Ret^lx/lx^) two weeks and three months after the MPTP treatment. In RET-deficient mice the DOPAC (A,C) and HVA (B,D) levels at both time-points are below the levels of saline-treated mice.

**Supplementary Figure 7: AAV5 injection leads to a persistent gliosis in the striatum but no inflammation in the striatum and no enrichment of astrocytes or microglia in the substantia nigra**

(A) Immunohistochemical staining of striatal sections for the astrocytic marker GFAP on the injected (inj.) and the contra-lateral side (con.) of control mice (DAT-Cre, Ret^lx/lx^) and RET-deficient mice (DAT-Ret^lx/lx^) two weeks and three months after the MPTP treatment. (B) The quantification reveals that in addition to the MPTP-induced transient gliosis observed at the two-week time point, there is also 4-fold lower gliosis at the three month time point (compared to the two week time point). This effect correlates with AAV5 injection, but is independent of the mouse genotype, the AAV5 encoded protein, and the virus injection injury. (C) Immunohistochemical staining of striatal sections for the microglia marker Iba-1 from the indicated mice, treatments and time points. (D) Immunohistochemical staining of SNpc sections for GFAP from the indicated mice, treatments and time points. (E) Immunohistochemical staining of SNpc sections for Iba-1 from the indicated mice, treatments and time points. Scale bar = 100 µm; n ≥ 3.
